# Polymorphisms in the Glucagon-Like Peptide 1 Receptor (*GLP-1R*) Gene Are Associated with the Risk of Coronary Artery Disease in Chinese Han Patients with Type 2 Diabetes Mellitus: A Case-Control Study

**DOI:** 10.1155/2018/1054192

**Published:** 2018-09-09

**Authors:** Xiaowei Ma, Ran Lu, Nan Gu, Xiaowei Wei, Ge Bai, Jianwei Zhang, Ruifen Deng, Nan Feng, Jianping Li, Xiaohui Guo

**Affiliations:** ^1^Department of Endocrinology, Peking University First Hospital, Beijing, China; ^2^Department of Cardiovascular, Peking University First Hospital, Beijing, China

## Abstract

**Background:**

Glucagon-like peptide 1 (GLP-1) bestows protective effects upon the cardiovascular system through direct cardiovascular interactions or by improvements to metabolic function. Both these effects are thought to be at least partly mediated by the GLP-1 receptor (GLP-1R). This case-controlled study investigated whether polymorphisms in the *GLP-1R* gene affect the risk of cardiovascular disease in type 2 diabetic patients in the Chinese Han population.

**Methods:**

Eleven haplotype-tagging single nucleotide polymorphisms (SNPs), distributed across 22 kb of the 39 kb *GLP-1R* gene, were selected and genotyped in diabetic patients from a Chinese Han population. Patients were classified based on the severity of coronary artery stenosis. Coronary artery stenosis was ≥50% in 394 patients (coronary artery disease- (CAD-) positive group), and coronary artery stenosis was <50% in 217 patients (control group). Allele and genotype frequencies were compared between the two groups at all 11 SNPs.

**Results:**

When considered in recessive inheritance mode, patients with the GG genotype at rs4714210 had a lower CAD risk than patients with other genotypes (OR = 0.442, 95% CI = 0.258–0.757, *p* = 0.002), even when other known CAD risk factors were taken into account (OR_a_ = 0.440, 95% CI_a_ = 0.225–0.863, *p*_a_ = 0.017). In additive inheritance mode, GG genotype carriers at rs4714210 exhibited a lower risk of CAD than AA carriers (OR_a_ = 0.475, CI_a_ = 0.232–0.970, *p*_a_ = 0.041).

**Conclusion:**

In type 2 diabetic patients from a Han Chinese population, some variations in the *GLP-1R* gene were associated with a lower risk of developing CAD.

## 1. Introduction

Coronary artery disease (CAD) is a life-threatening condition that is a frequently occurring complication in patients with type 2 diabetes mellitus (T2DM), with diabetic patients being 2–4 times more likely to develop CAD than nondiabetics [1]. Determination of genetic variants associated with CAD development in T2DM patients may assist in the identification of at-risk individuals and allow targeting of primary prevention and early intervention measures. In recent years, glucagon-like peptide 1 receptor (GLP-1R) agonists such as exenatide and liraglutide have been widely studied because of their glucose-dependent insulinotropic effects [2] and their other physiological effects such as decrease in fatty acid absorption, increase in satiety, and reduction in body weight [3]. GLP-1Rs are widely expressed in the cardiovascular system [4], and a number of beneficial effects that protect against coronary heart disease are associated with the GLP-1/GLP-1R signal pathway and its agonist interactions. Previous studies demonstrated that GLP-1 agonists could reduce the rate of the first occurrence of death from cardiovascular causes and nonfatal myocardial infarction among patients with T2DM [5, 6]. GLP-1 agonists were also found to improve heart function, decrease the size of infarct areas in ischemia-reperfusion heart models (pig and mouse) [7], increase coronary blood flow in isolated mouse heart [4], and reduce monocyte adhesion and atherosclerotic lesions in apoE^−/−^ mice [8]. Consequently, we reasoned that genetic variation in the *GLP-1R* gene might affect CAD risk in patients with T2DM. This hypothesis was investigated by examining CAD-positive and CAD-negative patients with T2DM in a Chinese Han population.

## 2. Materials and Methods

### 2.1. Ethics Statement

The study protocol and informed consent procedures were approved by the Research Ethics Committees of Peking University First Hospital. Written informed consents were acquired from all subjects participating in this study, in agreement with the 1975 Helsinki Declaration.

### 2.2. Subjects

Diabetes mellitus was diagnosed according to World Health Organization criteria (1999) [9] as follows: fasting plasma glucose ≥ 7.0 mmol/l, and/or 2 h plasma glucose ≥ 11.1 mmol/l, or casual plasma glucose (random blood sugar) ≥ 11.1 mmol/l. Patients with type 1 diabetes and subjects with active inflammatory conditions, autoimmune diseases, malignancies, usage of immunosuppressive drugs, and known hematological disorders were excluded. In total, 611 unrelated Chinese Han subjects with T2DM were included in the study: 394 with coronary artery stenosis (CAS) ≥ 50% (CAD-positive group) and 217 with CAS < 50% (control group). Diagnostic procedures were carried out at Peking University First Hospital. CAS ≥ 50% individuals were defined as those who exhibited ≥ 50% stenosis in at least one of the major coronary arteries or their main branches upon cardiac catheterization. Control individuals had <50% coronary stenosis in all main coronary arteries and main branches as determined by cardiac catheterization or high specificity spiral computed tomography (CT) scan [10]. Demographic data and patient cardiovascular risk factor data were collected for all subjects from medical records. These data comprised gender, age, body mass index (BMI), fasting plasma glucose (FPG), history of dyslipidemia, hypertension (blood pressure ≥ 140/90 mmHg or receiving any antihypertensive therapies), and smoking history (“ever” or “never,” with “ever” defined as having smoked more than one cigarette per day for more than 6 months, as per World Health Organization criteria).

### 2.3. Single Nucleotide Polymorphism Genotyping

Genomic DNA was extracted from peripheral blood using a Whole Blood DNA Extraction Kit (BioTeke).

The GLP-1R gene is located at chromosome 6p21, is 38.9 kb in length, and includes 13 exons. In total, 33 haplotype-tagging single nucleotide polymorphisms (SNPs) were identified at the GLP-1R locus in the CHB (Han Chinese from Beijing) population from the HapMap Phase II database (http://www.hapmap.org) (R#27, *r* ^2^ < 0.8, MAF ≥ 0.05). Eleven of these SNPs, dispersed across 22,058 bp of the total 38,964 bp of *GLP-1R*, were selected for further study: rs761387 (T>C), rs2268635 (G>A), rs7769547 (G>A), rs910162 (T>A), rs3765468 (G>A), rs3765467 (G>A), rs3765466 (A>T), rs10305456 (C>T), rs10305518 (T>G), rs1820 (T>A), and rs4714210 (A>G). Target regions were amplified by PCR. Direct DNA sequencing was used for 8 of the 11 selected SNPs, using a MassARRAY system (Sequenom iPLEX assay, San Diego, CA, USA) [11], and the remaining three SNPs were genotyped using polymerase chain reaction-restriction fragment length polymorphism (PCR-RFLP) analysis. Genotyping success rates were 95–100%, and repeatability rates were 98–100%. To validate PCR-RFLP assays, 5% of amplicons were directly sequenced to confirm the genotypes for each SNP. Concordance rates between RFLP and DNA-sequencing results were 98–100%.

### 2.4. Data Analysis

Clinical and laboratory data were expressed as means ± SD or percentages. Genotype distributions described departure from Hardy-Weinberg equilibrium at each polymorphic locus. Linkage disequilibrium (LD) and haplotype analysis were performed using Haploview 4.2, with haplotypes estimated using an accelerated expectation-maximization algorithm.

Allele frequencies were determined by gene counting. SNP association with risk of CAD was assessed using the SPSS statistical package (SPSS version 19.0, USA). Qualitative variables were compared using a *χ* ^2^ test, and quantitative variables were compared using an independent samples *t*-test or a Mann–Whitney *U* test. Associations between CAD and genotype were analyzed using multiple logistic regression with adjustment for the following potential confounders: age, gender, BMI, smoking status, positive histories of dyslipidemia and hypertension, and diabetic duration. As a descriptive measure of association between genotypes and outcomes, *p* < 0.05 was considered to be statistically significant and odds ratios (ORs) were calculated with 95% confidence intervals (CIs). Bonferroni correction was used to correct for multiple comparisons. Power and Sample Size Calculation software (version 3.1.2, 2014) was used for power calculations [12].

## 3. Results

### 3.1. Characteristics of Study Subjects

Males were more likely to be in the CAD-positive T2DM group than in the control T2DM group (*p* < 0.05); otherwise, no significant differences in phenotypic characteristics were found between groups (Table 1). HbA1c and FPG measurements were acquired after antidiabetic treatment. Genotype distributions at all 11 loci were in agreement with Hardy-Weinberg equilibrium (data not shown). Statistical power was 0.99.

### 3.2. Allele and Genotype Analysis

The minor allele G at rs4714210, in the 3′ untranslated region (UTR), was found more frequently in the control group than in the CAD-positive group, and carriers of the G allele displayed a lower risk of CAD when compared with noncarriers (OR = 0.783, 95% CI = 0.613–1.002, *p* = 0.051). In dominant inheritance mode, no significant difference in genotype distribution was found between the CAD-positive and control groups (Supplementary Material 1).

The protective effect of the homozygous GG minor allele genotype at rs4714210 was also observed in additive (codominant) inheritance mode using logistic regression analysis (Table 2). GG genotype carriers at rs4714210 exhibited a lower risk of CAD than AA carriers (OR_a_ = 0.475, CI_a_ = 0.232–0.970, *p*_a_ = 0.041, after adjustment for confounders as above), while no such protective effect was observed in heterozygote carriers.

In recessive inheritance mode, the carriers of genotype GG at rs4714210 had a decreased risk of CAD (OR = 0.442, 95% CI = 0.258–0.757, *p* = 0.002; OR_a_ = 0.440, 95% CI_a_ = 0.225–0.863, *p*_a_ = 0.017), after adjusting for other known CAD risk factors (gender, age, BMI, smoking status, dyslipidemia history, hypertension history, and diabetic duration) (Table 3).

The other ten SNPs tested in this study displayed similar allele frequencies between the CAD-positive and control groups, and no significant associations were noted between genotype and CAD risk (Tables 2 and 3).

### 3.3. Haplotype Analysis

Haploview plotting was used to construct haplotypes depending on the physical position and the value of *D*′ (*D*′ > 0.5) between each pair of SNPs in one block. Three blocks were delineated as follows: LD block 1 (rs910162, rs3765468, rs3765467, rs765466, and rs10305456), block 2 (rs761387 and rs7769547), and block 3 (rs10305518, rs1820, and rs4714210). The SNP distributions in the three haplotype blocks did not differ significantly between the CAD-positive and control groups (Supplementary Material 2).

## 4. Discussion

GLP-1R is a 463 amino acid member of the class B GPCR secretin family. GLP-1R is a classic seven-transmembrane protein; the C-terminus of GLP-1R interacts with a signaling G protein, and the large N-terminal extracellular domain plays an important role in ligand binding [13, 14]. After binding to the GLP-1 ligand, GLP-1R transmits a signal through a Gas-coupled subunit. This induces an increase in cAMP (cyclic adenosine monophosphate) levels and consequently activates the PKA pathway. The effect of the GLP-1/GLP-1R pathway in the myocardial ischemia-reperfusion model was previously summarized by Ravassa et al. [15]. In particular, GLP-1/GLP-1R can activate the PKA, PI3K, MEK1/2, and eNOS pathways, resulting in cardiovascular protective effects such as reduced apoptosis, improved energy metabolism, reduced inflammation in myocardial cells, and vasodilation in myocardial arteries. Systematically, GLP-1R transmits signals that prompt insulin secretion increases, appetite reduction, metabolism improvement, and lower blood pressure and, as a result, decreases the severity of atherosclerotic lesions [5]. The wide-ranging effects of GLP-1/GLP-1R suggest that variations in the *GLP-1R* gene may contribute to the risk of CAD.

Here, we found an association between rs4714210 in the *GLP-1R* gene and CAD risk in T2DM patients in the Chinese Han population. Patients homozygous for the minor allele G at rs4714210 exhibited a 50% lower risk of CAD than other genotype carriers. The mechanisms through which this allele confers protection are unclear. The rs4714210 locus is in the 3′ UTR of the *GLP-1R* gene, and 3′ UTRs are thought to play important roles in gene regulation. For example, 3′ UTRs can influence chromosome structure, regulate transcription, stabilize mRNA, and modulate translation, thus affecting the stability and transport of the encoded proteins [16]. We therefore speculate that variations at rs4714210 may differentially affect the function of GLP-1R through one or more of these mechanisms, but this remains to be confirmed. It is also likely that the rs4714210 SNP is in strong LD with other SNPs that have biological effects.

*GLP-1R* SNPs have been confirmed in the association with obesity [17], pancreatic beta-cell function [18, 19], and T2DM [20] in different populations. However, there are few studies about the variations of *GLP-1R* with CAD. In 2016, Scott et al. first observed an effect of GLP-1R genetic variation in Caucasian CAD patients with or without T2DM [21], identifying an association between *GLP-1R* rs10305492 and CAD in Caucasians (*p* < 0.05). Although SNP rs10305492 is not found in the Chinese Han population, SNPs rs10305492 and rs4714210 are in complete LD in Caucasians (*r* ^2^ = 1), indicating that our results are concordant with Scott's.

We acknowledge some limitations of this study. Sample size was relatively small, for in the cases were only 26 type 2 diabetes patients with one CAD vessel affected, 83 and 285 for two and three CAD vessels affected, respectively, so we did not stratify the cases and analyze the association with the number of affected vessels. And clinical features were not perfectly matched, and urine albumin creatinine ratio (ACR) was not collected, between the case and control groups. Both of them may introduce bias. Moreover, further functional studies on genetic variations at the *GLP-1R* locus would be beneficial. If our findings were confirmed through prospective studies, *GLP-1R* polymorphisms could be used as predictors of CAD risk in patients with T2DM in the Chinese Han population.

## 5. Conclusions

In T2DM patients from a Han Chinese population, some variations in the *GLP-1R* gene were associated with a lower risk of developing CAD.

## Figures and Tables

**Table 1:** Clinical characteristics of CAD-positive and control patients with T2DM.

	CAD-pos.	Controls	*p*
*N*	394	217	
Male, *n* (%)	274 (69.5)	97 (44.7)	<0.001
Age (y)	61.38 ± 10.13	62.19 ± 10.28	0.384
T2DM duration (y)	6.0 (1.0–12.0)	6.0 (2.0–10.0)	0.912
BMI (kg/m^2^)	26.1 ± 3.55	25.9 ± 3.65	0.521
HbA1c (%)	7.28 ± 1.45	7.09 ± 1.64	0.275
FPG (mmol/l)	7.3 ± 2.7	6.9 ± 2.1	0.164
eGFR (ml/min/1.73m^2^)	70.0 (58.94–81.72)	77.8 (57.9–85.0)	0.279
Positive dyslipidemia history (%)	76.5	78.1	0.697
Positive hypertension history (%)	79.3	72.8	0.106
Positive smoking history (%)	45.1	37.1	0.099

Data are presented as mean ± SD, *n* (%). BMI: body mass index; FPG: fasting plasma glucose; CAD: coronary artery disease; T2DM: type 2 diabetes mellitus. Independent *t*-test was used to compare BMI, HbA1c, FPG, and T2DM duration between groups; Mann–Whitney *U* test was used to compare the difference in T2DM duration between two groups. Age was compared by *t*-test. Other phenotypic characteristics were compared by *χ* ^2^ test between groups.

**Table 2:** Distribution of SNP genotype frequencies in CAD-positive and control groups in additive inheritance mode.

SNP	Genotype	CAD-pos. *n* = 394 (%)	Controls *n* = 217 (%)	*p*	OR_a_	CI_a_	*p*_a_
rs761387	TT	268 (68.0)	142 (65.4)		1		
	TC	113 (28.7)	69 (31.8)	0.696	0.855	0.530–1.382	0.523
	CC	13 (3.3)	6 (2.8)		1.065	0.277–4.102	0.927

rs2268635	GG	168 (42.6)	109 (50.2)		1		
	GA	186 (47.2)	88 (40.6)	0.193	1.064	0.666–1.699	0.795
	AA	40 (10.2)	20 (9.2)		1.105	0.496–2.458	0.807

rs7769547	GG	102 (25.9)	54 (24.9)		1		
	GA	208 (52.8)	107 (49.3)	0.446	0.829	0.476–1.445	0.509
	AA	84 (21.3)	56 (25.8)		0.750	0.390–1.442	0.388

rs910162	TT	91 (23.1)	57 (26.3)		1		
	TA	220 (55.8)	105 (48.4)	0.204	1.127	0.653–1.945	0.668
	AA	83 (21.1)	55 (25.3)		0.920	0.480–1.764	0.801

rs3765468	GG	268 (68.0)	143 (65.9)		1		
	GA	112 (28.4)	68 (31.3)	0.683	0888	0.548–1.441	0.632
	AA	14 (3.6)	6 (2.8)		1.201	0.317–4.546	0.788

rs3765467	GG	240 (60.9)	143 (65.9)		1		
	GA	134 (34.0)	65 (30.0)	0.469	0.883	0.553–1.411	0.603
	AA	20 (5.1)	9 (4.1)		2.546	0.557–11.64	0.228

rs3765466	AA	67 (17.0)	35 (16.1)		1		
	AT	204 (51.8)	108 (49.8)	0.766	0.681	0.349–1.330	0.261
	TT	123 (31.2)	74 (34.1)		0.833	0.401–1.731	0.625

rs10305456	CC	330 (83.8)	177 (81.6)		1		
	CT	63 (16.0)	39 (18.0)	0.743	1.114	0.611–2.030	0.725
	TT	1 (0.3)	1 (0.5)		/^a^	/	/

rs10305518	TT	281 (71.3)	155 (71.4)		1		
	TG	102 (25.9)	57 (26.3)	0.935	0.812	0.487–1.354	0.425
	GG	11 (2.8)	5 (2.3)		0.985	0.250–3.887	0.983

rs1820	TT	341 (86.5)	189 (87.1)		1		
	TA	49 (12.4)	28 (12.9)	0.170	1.165	0.584–2.324	0.665
	AA	4 (1.0)	0 (0.0)		/	/	/

rs4714210	AA	173 (43.9)	88 (40.6)		1		
	AG	193 (49.0)	97 (44.7)	**0.01**	1.159	0.715–1.878	0.550
	GG	28 (7.1)	32 (14.7)		0.475	0.232–0.970	**0.041**

*p* for the chi-square test using crosstabulation. ORs are odds ratios of each genotype as compared with homozygous for the major allele. Logistical regression was used to calculate ORs, CIs (95% confidence intervals of ORs), and corresponding *p* values (*p*_a_), and all three values are presented after adjustment for gender, age, BMI, smoking status, hyperlipidemia history, hypertension history, and diabetic duration. ^a^OR_a_, CI_a_, or *p*_a_ could not be acquired because allele frequencies were too small.

**Table 3:** Distribution of SNP genotype frequencies in CAD-positive and control groups in recessive inheritance mode.

SNPs	CAD-pos. *n* = 394 (%)	Controls *n* = 217 (%)	OR	95% CI	*p*	OR_a_	95% CI_a_	*p*_a_
rs761387								
CC	13 (3.3)	6 (2.8)	1.200	0.450–3.203	0.716	1.123	0.295–4.283	0.865
TX	381 (96.7)	211 (97.2)	1			1		
rs2268635								
AA	40 (10.2)	20 (9.2)	1.113	0.633–1.957	0.710	1.070	0.499–2.295	0.862
GX	354 (89.8)	197 (90.8)	1			1		
rs7769547								
AA	84 (21.3)	56 (25.8)	0.779	0.528–1.148	0.207	0.851	0.500–1.447	0.551
GX	310 (78.7)	161 (74.2)	1			1		
rs910162								
AA	83 (21.1)	55 (25.3)	0.786	0.532–1.161	0.226	0.847	0.497–1.446	0.544
TX	311 (78.9)	162 (74.7)	1			1		
rs3765468								
AA	14 (3.6)	6 (2.8)	1.296	0.491–3.421	0.600	1.249	0.333–4.682	0.742
GX	380 (96.4)	211 (97.2)	1			1		
rs3765467								
AA	20 (5.1)	9 (4.1)	1.236	0.553–2.764	0.605	2.662	0.588–12.04	0.204
GX	374 (94.9)	208 (95.9)	1			1		
rs3765466								
TT	123 (31.2)	74 (34.1)	0.877	0.617–1.248	0.466	1.130	0.691–1.848	0.627
AX	271 (68.8)	143 (65.9)	1			1		
rs10305456								
TT	1 (0.3)	1 (0.5)	0.550	0.034–8.831	1.000	/^a^	/	/
AX	393 (99.7)	216 (99.5)	1			1		
rs10305518								
GG	11 (2.8)	5 (2.3)	1.218	0.418–3.551	0.718	1.044	0.267–4.083	0.950
TX	383 (97.2)	212 (97.7)	1			1		
rs1820								
AA	4 (1)	0 (0)	0.990	0.980–1.000	0.303	/	/	0.999
TX	390 (99)	217 (100)	1			1		
rs4714210								
GG	28 (7.1)	32 (14.7)	0.442	0.258–0.757	**0.002**	0.440	0.225–0.863	**0.017**
AX	366 (92.9)	185 (85.3)				1		

CAD: coronary artery disease; OR: odds ratio; CI: confidence interval. OR_a_, CI_a_, and *p*_a_ represent OR, CI, and *p* after adjustment for gender, age, BMI, smoking status, hyperlipidemia history, hypertension history, and diabetic duration. OR, 95% CI, and *p* values were compared using chi-square analysis. OR_a_, 95% CI_a_, and *p*_a_ were assessed using multiple logistic regression analysis. ^a^OR_a_, CI_a_, or *p*_a_ could not be acquired because allele frequencies were too small.

## Supplementary Materials

Supplementary Materials Supplementary Material 1: distribution of SNP genotype frequencies in CAD-positive and control groups in dominant inheritance mode. Supplementary Material 2: association of common haplotypes with CAD risk.
