# Supplementary material for: Polymorphisms in the Glucagon-Like Peptide 1 Receptor (GLP-1R) Gene Are Associated with the Risk of Coronary Artery Disease in Chinese Han Patients with Type 2 Diabetes Mellitus: A Case-Control Study
Source: J Diabetes Res. 2018 Sep 9;2018:1054192. doi: 10.1155/2018/1054192 (PMC6151225; doi:10.1155/2018/1054192)
Supplement: Supplementary Materials — Supplementary Material 1: distribution of SNP genotype frequencies in CAD-positive and control groups in dominant inheritance mode. Supplementary Material 2: association of common haplotypes with CAD risk. [file 1054192.f1.pdf]

Supplementary Material 1

Distribution of SNP genotype frequencies in CAD-positive and control groups in dominant inheritance mode

SNPs	CAD-pos. n = 394(%)	Controls n = 217(%)	OR	95% CI	*P*	OR_a	95% CI_a	P_a
rs761387								
CX	126(32.0)	75(34.6)	0.890	0.627–1.264	0.516	0.871	0.546–1.390	0.563
TT	268(68.0)	142(65.4)	1			1		
rs2268635								
AX	226(57.4)	108(49.8)	1.358	0.973–1.894	0.071	1.071	0.684–1.676	0.765
GG	168(42.6)	109(50.2)	1			1		
rs7769547								
AX	292(74.1)	163(75.1)	0.948	0.648–1.389	0.785	0.804	0.474–1.363	0.418
GG	102(25.9)	54(24.9)	1			1		
rs910162								
AX	303(76.9)	160(73.7)	1.186	0.809–1.739	0.381	1.062	0.632–1.785	0.820
TT	91(23.1)	57(26.3)	1			1		
rs3765468								
AX	126(32.0)	74(34.1)	0.909	0.639–1.291	0.593	0.913	0.571–1.461	0.704
GG	268(68.0)	143(65.9)	1			1		
rs3765467								
AX	154(39.1)	74(34.1)	1.240	0.877–1.753	0.223	0.963	0.609–1.521	0.870
GG	240(60.9)	143(65.9)	1			1		
rs3765466								
TX	328(83.2)	182(83.9)	0.956	0.611–1.496	0.843	0.741	0.388–1.417	0.365
AA	66(16.8)	35(16.1)	1			1		
rs10305456								
TX	64(16.2)	40(18.4)	0.858	0.555–1.326	0.491	1.114	0.611–2.030	0.725
CC	330(83.8)	177(81.6)	1			1		
rs10305518								
GX	113(28.7)	62(28.6)	1.005	0.697–1.451	0.977	0.827	0.504–1.356	0.452
TT	281(71.3)	155(71.4)	1			1		
rs1820								
AX	53(13.5)	28(12.9)	1.049	0.642–1.715	0.848	1.202	0.604–2.392	0.600
TT	341(86.5)	189(87.1)	1			1		
rs4714210								
GX	221(56.1)	129(59.4)	0.871	0.623–1.220	0.422	0.963	0.613–1.514	0.870
AA	173(43.9)	88(40.6)	1			1		

CAD, coronary artery disease; OR, odds ratio; CI, confidence interval. OR_a, CI_a, and p_a represent OR, CI, and *p* after adjustment for gender, age, BMI, smoking status, dyslipidemia history, hypertension history, and diabetic duration. OR, 95% CI, and *p* values were compared using Chi-square analysis. OR_a, CI_a, and p_a were assessed with multiple logistic regression analysis.

Supplementary Material 2

Association of common haplotypes with CAD risk

Haplotype Block 1	rs910162	rs3765468	rs3765467	rs3765466	rs10305456	CAD-pos.(%)	Controls(%)	*P*
1	A	G	G	T	C	48.3	49.3	0.746
2	T	G	A	A	C	21.8	18.9	0.221
3	T	A	G	A	C	17.4	18.2	0.722
4	T	G	G	T	T	8.1	9.2	0.486
5	T	G	G	A	C	2.7	3.5	0.448
Block 2	rs761387	rs7769547						
1	T	A				47.1	50.0	0.336
2	T	G				35.2	31.3	0.168
3	C	G				17.1	18.2	0.612
Block 3	rs10305518	rs1820	rs4714210					
1	T	T	A			45.4	41.3	0.094
2	T	T	G			31.5	36.8	0.107
3	G	T	A			15.7	15.4	0.914
4	T	A	A			7.2	6.2	0.952

CAD, coronary artery disease. Chi-square analysis was used for statistical comparisons. *P* values were acquired by haplotype analysis using Haploview 4.2 software.
